# Significance of nested PCR testing for the detection of low-density malaria infection amongst febrile patients from the Malaria Elimination Demonstration Project in Mandla, Madhya Pradesh, India

**DOI:** 10.1186/s12936-022-04355-8

**Published:** 2022-11-17

**Authors:** Akansha Singh, Mrigendra P. Singh, Sneha Bhandari, Harsh Rajvanshi, Sekh Nisar, Vinay Telasey, Himanshu Jayswar, Ashok K. Mishra, Aparup Das, Harpreet Kaur, Altaf A. Lal, Praveen K. Bharti

**Affiliations:** 1 Indian Council of Medical Research - National Institute of Research in Tribal Health (ICMR-NIRTH), Jabalpur, Madhya Pradesh India; 2 Malaria Elimination Demonstration Project, Mandla, Madhya Pradesh India; 3 Indian Council of Medical Research - National Institute of Research in Environment Health (ICMR-NIREH), Bhopal, Madhya Pradesh India; 4 Directorate of Health Services, Government of Madhya Pradesh, Bhopal, India; 5 Department of Health Research, Indian Council of Medical Research, Ministry of Health and Family Welfare, New Delhi, India; 6 Foundation for Disease Elimination and Control of India, Mumbai, Maharashtra India; 7 Indian Council of Medical Research - National Institute of Malaria Research (ICMR-NIMR), New Delhi, India; 8 Present Address: Asia Pacific Leaders Malaria Alliance (APLMA), Sengkang, Singapore; 9 Present Address: Department of Health and Family Welfare, NHM Raigarh, Chattisgarh, India

**Keywords:** Malaria, Low-density infection, PCR, *P. falciparum*, *P. vivax*

## Abstract

**Background:**

Low-density malaria infections (LDMI) are defined as infections that are missed by the rapid diagnostic test (RDT) and/or microscopy which can lead to continued transmission and poses a challenge in malaria elimination efforts. This study was conducted to investigate the prevalence of LDMI in febrile cases using species-specific nested Polymerase Chain Reaction (PCR) tests in the Malaria Elimination Demonstration Project, where routine diagnosis was conducted using RDT.

**Methods:**

Every 10th fever case from a cross-sectional community based fever surveillance was tested with RDT, microscopy and nested PCR. Parasite DNA was isolated from the filter paper using Chelex based method. Molecular diagnosis by nested PCR was performed targeting 18SrRNA gene for *Plasmodium* species.

**Results:**

The prevalence of malaria was 2.50% (436/17405) diagnosed by PCR, 1.13% (196/17405) by RDT, and 0.68% (118/ 17,405) by microscopy. Amongst 17,405 febrile samples, the prevalence of LDMI was 1.51% (263/17405) (95% CI 1.33–1.70), which were missed by conventional methods. Logistic regression analysis revealed that illness during summer season [OR = 1.90 (p < 0.05)] and cases screened within three days of febrile illness [OR = 5.27 (p < 0.001)] were the statistically significant predictors of LDMI.

**Conclusion:**

The prevalence of malaria among febrile cases using PCR was 2.50% (436/17405) as compared to 1.13% (196/17405) by RDT. Higher number of the LDMI cases were found in subjects with ≤ 3 days mean duration of reported fever, which was statistically significant (p < 0.001). This observation suggests that an early detection of malaria with a more sensitive diagnostic method or repeat testing of the all negative cases may be useful for curtailing malaria transmission. Therefore, malaria elimination programme would benefit from using more sensitive and specific diagnostic methods, such as PCR.

## Background

Malaria in India is heterogeneous and complex disease because of its population, topography, epidemiology, and diverse climatic conditions. In 2020, globally, approximately 241 million malaria cases were detected. In the South East Asia region of the World Health Organization (WHO-SEARO), malaria case incidence has been reduced by 83% in the past two decades; from about 18 cases per 1000 population at risk in 2000 to about 3 cases in 2020 [1]. India contributes around 83% of cases in the South East Asia region and is progressing to eliminate malaria with the goal set by WHO as part of the global technical strategy, which aims zero indigenous malaria case by 2030 [2]. Furthermore, the National Centre for Vector Borne Diseases Control (NCVBDC) formerly known as National Vector Borne Disease Control Programme (NVBDCP) has developed the National Strategic Plan (NSP) for malaria elimination in a phased wise manner [3].

Malaria infection is mainly concentrated in the tribal dominated rural areas of Madhya Pradesh, Maharashtra, Odisha, Rajasthan, Gujarat, Jharkhand, Chhattisgarh, Andhra Pradesh, West Bengal, and Karnataka [4]. Elimination of malaria in India is challenging due to diverse climate, diverse vector population, epidemiology, emerging drug and insecticide resistance, migration of people, asymptomatic malaria, low density malaria infections (LDMI) and other technical and operational cause [5]. A LDMI is defined as an infection in which parasitaemia is missed by the conventional diagnostic methods, such as microscopy and rapid diagnostic test (RDT), but is identified by more sensitive Polymerase Chain Reaction (PCR) diagnostic tool [6]. The LDMI or sub-microscopic malaria infection presents a challenge for malaria elimination goal, because the individuals harbouring LDMI can continue to be sources of malaria transmission. These sub-microscopic infection often go undetected by microscopy/RDT as the threshold detection value of microscopy ranges approximately 40–100 parasites per µl of blood [7, 8].

In India, the sub-microscopic malaria burden has been previously reported [9–19]. Studies conducted so far using various molecular methods in different transmission settings, have reported the prevalence of sub-microscopic *P. falciparum* from 5 to 50% [20]. Hence, PCR testing remains the gold-standard for detecting these LDMI, because of its ability to detect parasitaemia as low as 1 parasite/µl (one gene copy per reaction) [7]. The present study was undertaken to determine the prevalence of LDMI among fever cases as part of the Malaria Elimination Demonstration Project (MEDP) using species-specific nested PCR tests.

## Methods

This study is a part of Malaria Elimination Demonstration Project, which is a first-of-its-kind public–private-partnership between the Indian Council of Medical Research (ICMR) through the National Institute for Research in Tribal Health (NIRTH) Jabalpur, Government of Madhya Pradesh (GoMP), and the Foundation for Disease Elimination and Control of India (FDEC-India, established by Sun Pharmaceutical Industries Ltd. as a not-for-profit entity) [21].

### Study site

The study was conducted in 1233 villages spread across nine blocks of district Mandla in Madhya Pradesh (MP), India. Mandla is geographically located (coordinates: 22°02′ and 23°22′ N latitudes, 80°18′ and 81°50′ E longitudes) in the east-central region and is a part of the Jabalpur division with a maximum part lying along the basin of river Narmada. Mandla has an area of 8771 km^2^, it is a densely forested district, inhabited by tribal community mainly ‘Gonds’ and ‘Baiga’ tribes with an estimated population of 11,43,126 [22].

### Sampling method

A cross sectional active fever surveillance was conducted fortnightly (7–14 days) from door to door by the trained Village Malaria Workers (VMWs), using T4 strategy (track-test-treat and track) [5]. The VMWs collected information on presenting symptoms related to malaria such as history of fever during last two weeks preceding the survey, fever on the day of survey, chills and rigor, headache, body ache, vomiting, number of days of onset of fever. During the active fever survey, bivalent malaria RDT (SD Bioline Malaria Ag Pf/Pv) was performed on every fever case for on-spot diagnosis and all the positive cases were treated with anti-malarials as per the national treatment guidelines.

**Fig. 1:**
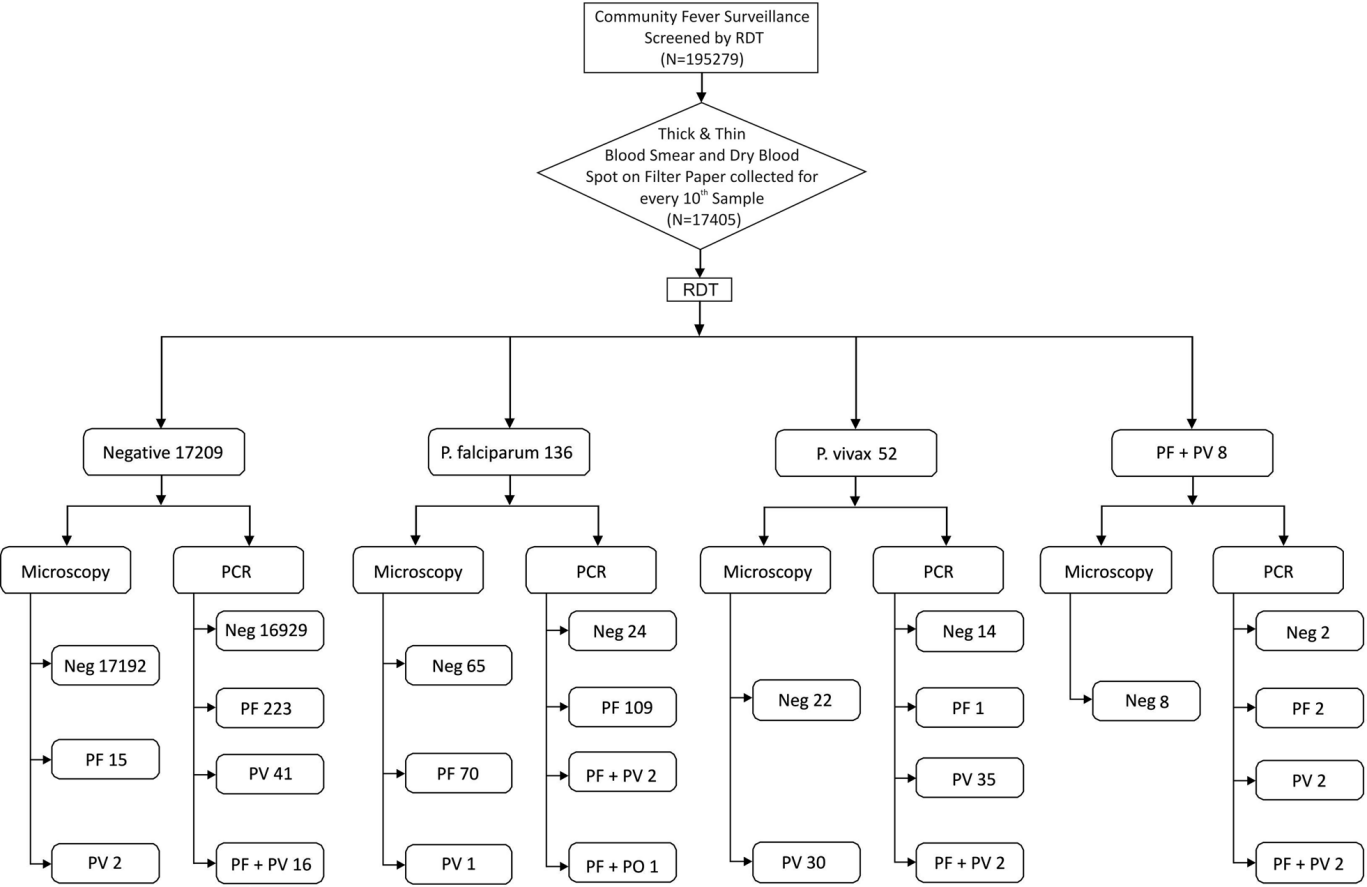
Flow chart showing distribution of cases diagnosed by Rapid Diagnostic Test, Microscopy and Polymerase Chain Reaction

Using systematic random sampling method, thick and thin blood smear was prepared for every 10th fever case on pre-cleaned glass slide; air-dried and was stored in slide boxes for further examination during January 2018 to December 2019. At the same time blood spot were also collected from finger pricks on 3 mm filter paper (Whatman, Maidstone, UK), air dried and stored in separate zip pouch with desiccant following appropriate laboratory protocols for molecular diagnosis using nested PCR. The samples were transported on monthly basis from district Mandla to the molecular parasitology laboratory at ICMR-NIRTH Jabalpur for further analysis (Fig. 1).

### Sample size

The sample size was determined using following formula of simple random sampling for finite population.

$$n=\frac{\frac{{z}^{2}p\left(1-p\right)}{{e}^{2}}}{1+(\frac{{z}^{2}p\left(1-p\right)}{{e}^{2}N})}.$$

The assumptions of 1% probability of LDMI in febrile patients with 25% relative precision were considered for getting an adequate sample size at 95% confidence limit. The population was taken 1,100,000 of the district population. Further, the derived number was multiplied with 1.5 as design effect and 40% inflated as non-response. This was guided to conduct study in 17,550 samples.

### Microscopic detection of malaria parasite

Both thick and thin blood smear were stained using JSB solution for microscopic examination. When no asexual parasites were observed after examination of 100 fields containing at least 10 white blood cells (WBCs) per field, a blood slide was considered negative. All the blood smears were re-examined by WHO certified Level-I microscopist at ICMR-NIRTH, who was blinded with the RDT result and results of first microscopy. Quantification of malaria parasite per 200 WBCs were performed on JSB stained thick blood films and parasite density was determined by calculating the number of asexual parasite × 6000/number of WBC counted as per WHO guidelines [23, 24].

### Molecular diagnosis

Genomic DNA was isolated from the dried blood spots (collected during Jan 2018 to Dec 2019) using Chelex method [Chelex-100 Sodium form (50–100 Mesh) Himedia Laboratories]. In brief, 3 punches of filter paper were cut and soaked in 1 ml of 0.5% saponin in phosphate buffered saline (PBS), vortexed for 20–30 s and incubated overnight at 4 °C. After incubation tubes were centrifuged at 3000 rpm for 60 s, fluid was aspirated and the filter paper was crushed in 100 µl of Mili Q water. After this step 50 µl of a stock solution of 20% Chelex-100 was added to these 1.5 ml tubes and heated at 95–99 °C in a heating block for 12 min and vortexed for every 2–3 min. The tube was centrifuged at 8000 rpm for 5 min the supernatant recovered, further the tube was centrifuged under the same condition for 10 min. the supernatant was collected in a new tube and stored at − 20 °C for further process [25]. The presence of *Plasmodium* species was determined using species specific nested PCR by targeting 18Sr RNA gene. To set up the primary PCR, 5 µl of genomic DNA as template was taken for the amplification of 18S rRNA gene for *Plasmodium* genus using forward and reverse primer. The primary PCR product was diluted 1:10 times and used for the nested PCR which was performed using four different species-specific primer pairs for *Plasmodium falciparum, Plasmodium vivax, Plasmodium malariae* and *Plasmodium ovale.* The primary PCR reaction was performed with 1× reaction buffer, 2 mM MgCl_2_, 0.2 mM dntp, 0.32 µM each primer and 0.75 U of Taq DNA polymerase for 25 µl reaction volume [Taq DNA Polymerase (Recombinant) 5 U/µl Genetix Biotech Asia Pvt. Ltsd] [26]. The details of PCR primers and cycling condition is given in Table 1. The PCR product was analysed on 1.2% Agarose gel electrophoresis. The only limitation of the present nested PCR study is that unlike qPCR, it didn’t quantify the parasites using the qPCR, which can correlate with the transmission of the parasites. However, nested PCR is able to detect one gene copy per reaction or a single parasite in the blood sample spotted on the filter paper.

**Table 1:** Primer sequence and PCR condition used for amplification of *Plasmodium* species

Genus/species	Primer name	Primer sequence	PCR product length (bp)	Denaturation	Annealing	Elongation	No of cycles
*Plasmodium*	F	TTAAAATTGTTGCAGTTAAAACG	1200 bp	95 °C, 1 min	53 °C, 0.75 min	72 °C, 1.5 min	35
	R	CCTGTTGTTGCCTTAAACTTC					
*P. falciparum*	F	TTAAACTGGTTTGGGAAAACCAAATATATT	205 bp	95 °C, 1 min	58 °C, 0.75 min	72 °C, 1 min	35
	R	ACACAATGAACTCAATCATGACTACCCGTC					
*P. vivax*	F	CGCTTCTAGCTTAATCCACATAACTGATAC	112 bp	95 °C, 1 min	60 °C, 1 min	72 °C, 1 min	35
	R	ACTTCCAAGCCGAAGCAAAGAAAG TCCTTA					
*P. malariae*	F	ATAACATAGTTGTACGTTAAGAATAACCGC	144 bp	95 °C, 1 min	58 °C, 0.75 min	72 °C, 1 min	35
	R	AAAATTCCCATGCATAAAAAATTATACAAA					
*P. ovale*	F	ATCTCTTTTGCTATTTTTTAGTATTGGAGA	800 bp	95 °C, 1 min	60 °C, 1 min	72 °C, 1 min	35
	R	GGAAAAGGACACATTAATTGTATCCTAGTG					

### Statistical analysis

The demographic (age, gender, and area of residence) variables, clinical symptoms related to malaria, results of RDT, microscopy, and PCR were entered in Microsoft Excel 2007 worksheet and numerically coded data was exported in R 4.1.2 for Windows (R Foundation for Statistical Computing, Vienna, Austria.) for statistical analysis. Sensitivity, specificity, positive predictive value (PPV), negative predictive value (NPV) with a 95% confidence interval of Microscopy and RDT against PCR as gold standard were calculated. The sensitivity was calculated as the number of true positives/ (true positives + false negatives), the specificity as the number of true negatives/ (true negatives + false positives), the PPV as the number of true positives/ (true positives + false positives) and the NPV as the number of true negatives/(true negatives + false negatives). Logistic regression analysis is used to examine the association of independent variable(s) with LDMI.

## Results

A total of 195,279 febrile cases were screened using RDT, out of which 17,405 samples were screened by light microscopy and PCR. The prevalence of malaria was 2.50% (436/17405) diagnosed by PCR, 1.13% (196/17,405) by RDT and 0.68% (118/17,405) by microscopy. Out of these 17,405 febrile cases, 196 cases (136 *P. falciparum*, 52 *P. vivax* and 8 mixed infection of *P. falciparum* + *P. vivax*) were found positive by RDT. Amongst the RDT negative cases (17,209), 17 cases (0.1%) and 280 cases (1.63%) were found positive by microscopy and PCR, respectively. Further, out of the 136 *P. falciparum* positive cases by RDT, 70 (51.47%) cases tested positive by microscopy and 109 (80.14%) tested positive by PCR.

However, one case was diagnosed as *P. vivax* by microscopy and three mixed *P. falciparum* + *P. vivax* + *P. ovale* by PCR diagnosis. At the same time, out of the 52 *P. vivax* cases diagnosed by RDT, 30 (57.69%) and 35 (67.30%) were also confirmed as *P. vivax* by microscopy and PCR, respectively. However, one case was diagnosed as *P. falciparum* and 2 were found to be mixture of *P. falciparum* and *P. vivax* using PCR (Fig. 1).

Out of the 8 mixed infections of *P. falciparum* + *P. vivax* diagnosed by RDT, PCR revealed two *P. falciparum,* two *P. vivax*, two mixed infections *(P. falciparum and P. vivax)*, and two negative cases. None of these were found positive by microscopy (Fig. 1).

**Fig. 2:**
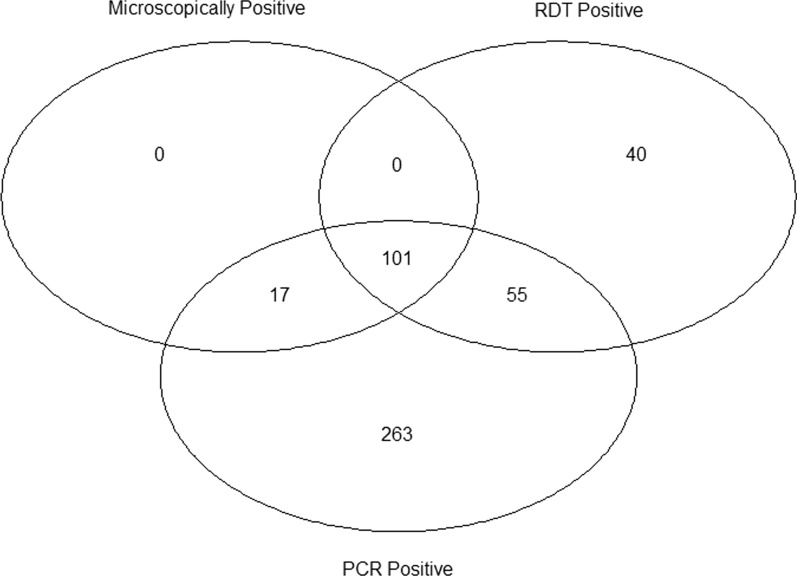
Venn diagram showing distribution of malaria positive cases found in different diagnostic methods

The malaria prevalence diagnosed either by RDT, microscopy or PCR was 2.73% (476/17405). Out of the 476 malaria cases, 101 (21.22%) cases were found positive by all three diagnostic methods. Seventeen of 476 cases were positive by microscopy and PCR, but negative by RDT. There were 55 cases positive by RDT and PCR but negative by microscopy (Fig. 2). Out of 17,405 febrile cases, 263 were positive only by PCR. Hence, the prevalence of LDMI was 1.51% (95% CI 1.33–1.70).

The monthly trend of malaria prevalence indicated highest number of cases in the month of March and lowest in the month of June. Whereas LDMI was found higher during April–May and least in the month of February (Table 2). A declining trend of malaria prevalence was observed with age however, there was no such trend showed in LDMI (Table 3).

**Table 2:** Monthly prevalence of malaria and low-density infection in active fever surveillance diagnosed by RDT, microscopy and PCR

Months	Fever cases screened	RDT positive (%)	Microscopy positive (%)	Gametocyte (%)	PCR positive (%)	Total malaria positive^a^ (%)	LDMI prevalence^b^ (%)	LDMI proportion^c^ (%)
January	904	8 (0.88)	7 (0.77)	5 (71.42)	34 (3.76)	35 (3.87)	26/904 (2.88)	26/35 (74.29)
February	928	16 (1.72)	10 (1.08)	0	16 (1.72)	23 (2.48)	6/928 (0.65)	6/23 (26.09)
March	1907	44 (2.31)	24 (1.26)	7 (29.16)	81 (4.25)	90 (4.72)	42/1907 (2.20)	42/90 (46.67)
April	853	8 (0.94)	4 (0.47)	0	26 (3.05)	26 (3.05)	15/853 (1.76)	15/26 (57.69)
May	865	3 (0.35)	3 (0.35)	0	25 (2.89)	25 (2.89)	21/865 (2.43)	21/25 (84.00)
June	1061	6 (0.57)	4 (0.38)	4 (100.00)	10 (0.94)	10 (0.94)	4/1061 (0.38)	4/10 (40.00)
July	1489	22 (1.48)	13 (0.87)	3 (23.07)	34 (2.28)	36 (2.42)	13/1489 (0.87)	13/36 (36.11)
August	2188	39 (1.78)	21 (0.96)	4 (19.04)	48 (2.19)	59 (2.70)	17/2188 (0.78)	17/59 (28.81)
September	2624	11 (0.42)	8 (0.30)	4 (50.00)	64 (2.44)	65 (2.48)	54/2624 (2.06)	54/65 (83.08)
October	2093	20 (0.96)	12 (0.57)	3 (25.00)	48 (2.29)	52 (2.48)	31/2093 (1.48)	31/52 (59.62)
November	1426	10 (0.70)	4 (0.28)	1 (25.00)	31 (2.17)	35 (2.45)	25/1426 (1.75)	25/35 (71.43)
December	1067	9 (0.84)	8 (0.75)	5 (62.50)	19 (1.78)	20 (1.87)	9/1067 (0.84)	9/20 (45.00)
Total	17,405	196 (1.13)	118 (0.68)	36 (30.50)	436 (2.50)	476 (2.73)	263/17405 (1.51)	263/476 (55.25)

^a^Total malaria positive by either or any diagnostic method i.e., RDT, microscopy or PCR

^**b**^Low density malaria infection/Fever cases screened

^**c**^Low density malaria infection/total malaria positive

**Table 3:** Age group wise prevalence of malaria and low-density infection in active fever surveillance diagnosed by RDT, microscopy and PCR

Months	Fever cases screened	RDT positive (%)	Microscopy positive (%)	Gametocyte (%)	PCR positive (%)	Malaria positive^a^ (%)	LDMI prevalence^b^ (%)	LDMI proportion^c^ (%)
0–1 yr	90	4 (4.44)	4 (4.44)	2 (50.00)	8 (8.89)	8 (8.89)	4/90 (4.44)	4/8 (50.00)
1–4 yrs	543	8 (1.47)	6 (1.10)	3 (50.00)	18 (3.31)	18 (3.31)	10/543 (1.84)	10/18 (55.56)
4–8 yrs	774	9 (1.16)	8 (1.03)	2 (25.00)	21 (2.71)	21 (2.71)	10/774 (1.29)	10/21 (47.62)
8–14 yrs	1331	11 (0.83)	6 (0.45)	4 (66.66)	20 (1.50)	22 (1.65)	10/1331 (0.75)	10/22 (45.45)
15+ yrs	14,667	164 (1.12)	94 (0.64)	25 (26.59)	369 (2.52)	407 (2.77)	229/14667 (1.56)	229/407 (56.27)
Total	17,405	196 (1.13)	118 (0.68)	36 (30.50)	436 (2.50)	476 (2.73)	263/17405 (1.51)	263/476 (55.25)

^a^Total malaria positive by either or any diagnostic method i.e., RDT, microscopy or PCR

^**b**^Low density malaria infection/Fever cases screened

^**c**^Low density malaria infection/total malaria positive

Gametocytes were detected in 30.50% (36/118) microscopically positive individuals. Whereas age group wise prevalence showed that the percentage of gametocytes was highest 67% (4/6) among 8–14 years of age group (Tables 2 and 3).

Using PCR as gold standard, the sensitivity and specificity of RDT was 35.8% and 99.8%, respectively. However, the sensitivity and specificity of microscopy was 27.1% and 100%, respectively, against PCR as the gold standard. In the case of *P. falciparum,* the sensitivity and specificity of RDT and microscopy against PCR was 32.5%, 99.8%, and 25.1%, 100% respectively. However, the sensitivity and specificity of RDT and microscopy for *P. vivax* was 44.9%, 99.9%, and 38.5%, 100%, respectively (Table 4).

**Table 4:** Diagnostic performance of RDT, and microscopy in reference to PCR as gold standard

Malaria positive	Malaria positive		*P. falciparum*		*P. vivax*	
	RDT	Microscopy	RDT	Microscopy	RDT	Microscopy
True positive	156	118	109	84	35	30
False negative	280	318	226	251	43	48
False positive	40	0	27	1	17	3
True negative	16,929	16,969	17,043	17,069	17,310	17,324
Sensitivity (95%CI)	35.8 (31.3–40.5)	27.1 (22.9–31.5)	32.5 (27.5–37.8)	25.1 (20.5–30.1)	44.9 (33.6–56.6)	38.5 (27.7–50.2)
Specificity (95%CI)	99.8 (99.7–99.8)	100 (100–100)	99.8 (99.8–99.9)	100 (100–100)	99.9 (99.8–99.9)	100 (99.9–100)
PPV (95%CI)	79.6 (73.3–85.0)	100 (96.9–100)	80.1 (72.4–86.5)	98.8 (93.6–100)	67.3 (52.9–79.7)	90.9 (75.7–98.1)
NPV (95%CI)	98.4 (98.2–98.6)	98.2 (97.9–98.4)	98.7 (98.5–98.9)	98.6 (98.4–98.7)	99.8 (99.7 -99.8)	99.7 (99.6–99.8)

CI, confidence interval; PPV, positive predictive value; NPV, negative predictive value

Bivariate logistic regression analysis revealed that the cases that had illness during summer season [OR = 1.90 (95% CI 1.02–3.54; p < 0.05)] and screened within three days of febrile illness [OR = 5.27 (95% CI 3.55–7.82; p < 0.001)] were the statistically significant predictors of LDMI. The multivariate logistic regression analysis showed that the cases screened within three days of febrile illness was a highly significant predictor of LDMI [(aOR = 5.17 (95% CI 3.47–7.70; p < 0.001)], with the controlled effect of all other independent factors such as endemicity, season, and age (Table 5).

**Table 5:** Logistic regression analysis of factors associated with low density infection of malaria

Factors	n/d (%)	cOR (95% CI)	aOR (95% CI)
Area of residence			
Low endemic	79/145 (54.48)	1 (reference)	1 (reference)
High endemic	184/331 (55.59)	1.05 (0.71–1.55)	1.04 (0.68–1.61)
Age group			
Child	24/47 (51.06)	0.83 (0.45–1.52)	0.96 (0.49–1.87)
Adult	239/429 (55.71)	1 (reference)	1 (reference)
Season			
Spring	74/148 (50.00)	1 (reference)	1 (reference)
Summer	40/61 (65.57)	1.90 (1.02–3.54)*	1.47 (0.75–2.87)
Monsoon	84/160 (52.50)	1.11 (0.71–1.73)	0.99 (0.60–1.62)
Winter	65/107 (60.75)	1.55 (0.93–2.56)	1.44 (0.83–2.50)
Duration of febrile illness			
≤ 3 days	197/274 (71.90)	5.27 (3.55–7.82)***	5.17 (3.47–7.70)***
> 3 days	66/202 (32.67)	1 (reference)	1 (reference)

n/d, numerator/denominator; cOR, crude odds ratio; CI, confidence intervals; aOR, adjusted odds ratio

*p < 0.05: level of significance

**p < 0.01: level of significance

***p < 0.001: level of significance

## Discussion

As India progresses towards achieving the goal of malaria elimination by 2030, low-density parasitemia may pose a threat. Although malaria is primarily diagnosed by RDT and microscopy in India, there are several limitations with their diagnostic performance. The RDT targets the histidine-rich protein-2 (HRP-2) of *P. falciparum,* and this target has limitations because of deletion in the natural parasite population and longer persistence of HRP-2 in the blood [27, 28].

Similarly, microscopy can have limitations, such as only properly trained microscopists can detect low density parasitaemia and mixed-species infections [19, 29]. Therefore, malaria cases that are missed by RDT or microscopy will remain untreated and may serve as reservoirs for seeding new infections and outbreaks. In comparison, species-specific sensitive diagnostic tools such as PCR provide an opportunity to detect cases that are missed by conventional tools of RDT and microscopy.

The present study was designed to determine the prevalence of LDMI amongst the fever cases in district Mandla of Madhya Pradesh, India using nested PCR tests. The study revealed that the malaria prevalence was 2.73% (476/17405) in febrile cases by using all three diagnostic methods. Similar low levels of malaria positivity in fever survey was reported in a previous study where RDT was used as the sole diagnostic tool under the T4 strategy [5].

Currently, RDT is considered as the principal diagnostic tool for community-based diagnosis of malaria in India. In the present study, only 1.13% (196/17,405) malaria positivity was recorded by RDT, whereas 2.50% (436/17405) positivity was recorded by PCR. There were 17 cases diagnosed positive by microscopy and PCR, but negative by RDT. This could be due to *Pfhrp2/3* gene deletion resulting in a false negative result. Others studies have reported *Pfhrp2* gene deletion in the entire country [27, 30].

Out of 476 malaria positive cases, 40 (8.40%) were found positive by RDT and missed by both PCR and microscopy, which could be due to circulation of Pfhrp2 in the blood from past infections. It has been previously reported by Kyabayinze et al. [31] that HRP2 antigen can persist for four weeks or longer in blood leading to false positive result, especially in high endemic areas. Moreover, this study also revealed that 11.55% (55/476) cases that were positive by PCR and RDT were missed by microscopy, which could be due to previously documented limitations of microscopy [32].

Out of the total prevalence of malaria (2.73%) amongst the febrile cases, more than half (1.51%) were LDMI that were only detected by PCR. Similar study conducted in two districts of Chhattisgarh between 2007 and 2008 showed that 66.3% of peripheral and 64.4% of placental infections were submicroscopic [9]. Another survey conducted during 2012–2015 at three sites in India (Chennai, Rourkela, and Nadiad) detected a high burden of submicroscopic infections of 71% in Chennai, 31% in Rourkela and 21.4% in Nadiad using PCR [11]. A study carried out from February 2017 to April 2017 during non-transmission season in two north-eastern districts of India showed low-levels of prevalence of LDMI (5.1%) [33].

In another high endemic area of Kandhamal district in Odisha, 18% *Plasmodium* infections were reported amongst the asymptomatic individuals by qPCR with 37% submicroscopic malaria [13]. The importance of nested PCR was also demonstrated by Noordin et al. in Malaysia [6] and Ethiopia [34]. Considering the limitations of microscopy and RDT, it is recommended that more sensitive diagnostic tool such as PCR may be deployed in routine surveillance to detect low-density parasite infections during the malaria elimination phase.

We observed a high prevalence of LDMI during the dry season (April–May) which is consistent with the findings of Dielmo and Ndiop in Senegal [35]. This study in Senegal concluded that when transmission reaches very low level, the traditional methods such as RDT/microscopy are inadequate to assess the scale of parasite reservoir [35]. The number of LDMI cases in the high endemic area were higher in comparison to the low endemic area, however, the difference was not significant statistically (p > 0.05). Studies have indicated that this observation may be due to the partially acquired immunity by individuals who live in high endemic areas and are repeatedly exposed to malaria [36, 37].

In the present study, it was observed that LDMI was likely to be associated with early febrile illness within 3 days. Early-stage malaria infection can be missed by microscopy and RDT as the sensitivity of microscopy and RDT is considerably lower (in the range of 40–100 parasites per µl of blood for microscopy and approximately 100 parasites per µl of blood in approximately 5 µl of whole blood for RDTs) [38, 39]. A study by Aninagyei et al.revealed that repeat testing in 12 h post-first test by microscopy improved the diagnostic efficacy by 12% [40].

Other studies have also reported an association of sub-microscopic malaria with fever and non-febrile illness [41, 42]. In this study, the highest prevalence of gametocytes was observed during dry season and in age group of 8–14 years, which suggests that there is perennial transmission of malaria in the community. The results of this study reveal that in comparison to RDT and microscopy, nested PCR detects approximately twice as many infections. Since, the PCR is a highly sensitive diagnostic method and can detect parasitaemia as low as one gene copy per reaction or a single parasite in the blood sample, it might be useful to deploy PCR based diagnostic tools in a sub-set of cases in surveillance to detect LDMI [7, 43].

As compared to microscopy and rapid diagnostic tests, the PCR methods are used in malaria surveillance as well as epidemiological research [44]. The use of dry blood spot on filter papers has allowed the use of PCR as the spots are easier to collect, store, and transport than EDTA whole blood in field settings [45]. In a recent meta-analysis by Naing et al., it was found that RDTs and microscopy have limited sensitivity and are inappropriate for the detection of asymptomatic/low density *Plasmodium* infections [46]. From a programmatic perspective as was done recently for COVID-19 and is also a practice for tuberculosis, the PCR-based diagnostic tests of malaria can be conducted in sentinel laboratories that would receive blood spotted filter paper from field sites. While this study has revealed the presence of sub-microscopic or sub-RDT infections that were revealed by PCR, it may be advisable to assess true burden of LDMI at community level through testing of both febrile cases and afebrile cases.

## Conclusion

This study has revealed that RDT and microscopy miss a significant number of low-density malaria infections. Though the approved and available RDT/microscopy-based diagnosis will stay as the diagnostic test of choice in case management of malaria, the results of this study suggests that PCR-based diagnostic tests should be used in malaria elimination programmes to determine true case-load of malaria among the febrile cases from high transmission areas. The present WHO guidance is to use RDTs and microscopy for malaria diagnosis, however, they also recommend use of PCR on a research-mode to develop a body of evidence for its use in surveillance. This study provides useful data for malaria elimination programmes to consider.

## Data Availability

We have reported all the findings in this manuscript. The hardcopy data is stored at MEDP data repository. If anyone wants to review or use the data, they should contact: Dr. Altaf A. Lal, Project Director—Malaria Elimination Demonstration Project, Mandla. Foundation for Disease Elimination and Control of India, Mumbai, India 482003. E-mail: altaf.lal@sunpharma.com, altaf.lal@gmail.com.
